# Accelerating read mapping with FastHASH

**DOI:** 10.1186/1471-2164-14-S1-S13

**Published:** 2013-01-21

**Authors:** Hongyi Xin, Donghyuk Lee, Farhad Hormozdiari, Samihan Yedkar, Onur Mutlu, Can Alkan

**Affiliations:** 1 Depts. of Computer Science and Electrical and Computer Engineering, Carnegie Mellon University, Pittsburgh, PA, 15213, USA; 2 Dept. of Computer Science, University of California Los Angeles, Los Angeles, CA, 90095, USA; 3 Dept. of Computer Engineering, Bilkent University, Ankara, 06800, Turkey

## Abstract

With the introduction of next-generation sequencing (NGS) technologies, we are facing an exponential increase in the amount of genomic sequence data. The success of all medical and genetic applications of next-generation sequencing critically depends on the existence of computational techniques that can process and analyze the enormous amount of sequence data quickly and accurately. Unfortunately, the current read mapping algorithms have difficulties in coping with the massive amounts of data generated by NGS.

We propose a new algorithm, FastHASH, which drastically improves the performance of the seed-and-extend type hash table based read mapping algorithms, while maintaining the high sensitivity and comprehensiveness of such methods. FastHASH is a generic algorithm compatible with all seed-and-extend class read mapping algorithms. It introduces two main techniques, namely *Adjacency Filtering*, and *Cheap K-mer Selection*.

We implemented FastHASH and merged it into the codebase of the popular read mapping program, mrFAST. Depending on the edit distance cutoffs, we observed up to 19-fold speedup while still maintaining 100% sensitivity and high comprehensiveness.

## Introduction

Massively parallel sequencing, or so-called next-generation sequencing (NGS), technologies have substantially changed the way biological research is performed since 2000 [1]. With these new DNA sequencing platforms, we can now investigate human genome diversity between populations [2], find genomic variants that are likely to cause diseases [3-8], and investigate the genomes of the great ape species [9-14] and even ancient hominids [15,16] to understand our own evolution. Despite all the revolutionary power these new sequencing platforms offer, they also present difficult computational challenges due to 1) the massive amount of data produced, 2) shorter read lengths, resulting in more mapping locations and 3) higher sequencing errors when compared to the traditional capillary-based sequencing.

With NGS platforms, such as the popular Illumina platform, billions of raw short reads are generated at a fast speed. Each short read represents a contiguous DNA fragment (i.e., 100 base-pairs (bp)) from the sequencing subject. After the short reads are generated, the first step is to *map *(i.e., align) the reads to a known reference genome. The mapping process is computationally very expensive since the reference genome is very large (e.g., the human genome has 3.2 gigabase-pairs). The software performing the mapping, called the mapper, has to search (query) a very large reference genome database to map millions of short reads. Even worse, each short read may contain *edits *(base-pairs different from the reference fragment, including mismatches, insertions and deletions) which requires expensive approximate searching. In addition, the ubiquitous common repeats and segmental duplications within the human genome complicate the task since a short read from such a genome segment corresponds to a large number of mapping locations in the reference genome.

To simplify searching a large database such as the human genome, previous work has developed several algorithms that fall into one of the two categories: *seed-and-extend *heuristic methods and *suffix-array *mapping methods.

The *seed-and-extend *heuristic is developed based on the observation that for a correct mapping, the short query read and its corresponding reference fragment, which is the piece of the reference genome that the query read should map to, must share some brief regions (usually 10-100 base-pair-long) of exact or inexact matches. These shorter shared regions, which indicate high similarity between the query read and the reference fragment, are called seeds. By identifying the seeds of a query read, the mapper narrows down the searching range from the whole genome to only the neighborhood region of each seed. Seeds are generated by preprocessing the reference genome and storing the locations of their occurrences in the reference genome in a separate data structure. During mapping, a seed-and-extend mapper first analyzes the query read to identify the seeds. Then, the mapper tries to extend the read at each of the seed locations via dynamic programming algorithms such as the Smith-Waterman [17] or Neddleman-Wunsch [18] algorithm.

On the other hand, the *suffix-array *mapping methods analyze the reference genome and transfer the reference genome into a suffix-array data structure, which mimics a suffix-tree of the reference genome. Each edge of this suffix-tree is labeled with one of the four base-pair types and each node containing all occurrence locations of a suffix. Walking through the tree from the root to leaf while concatenating all the base-pairs on the edges along the path together forms a unique suffix of the reference genome. Every leaf node of the tree stores all mapping locations of this unique suffix in the reference genome. Searching for a query read is equivalent to walking through the reference suffix-tree from the root to a leaf node following the query read's sequence. If there exists a path from the root to a leaf such that the corresponding suffix of the path matches the query read, then all the locations stored in the leaf node are returned as mapping locations. Suffix array uses the Burrows-Wheeler Transform [19] and the Ferragina-Manzini index [20] to mimic the suffix-tree traversal process with much smaller memory footprint.

Several mappers have been developed over the past few years. These mappers can be classified into two categories based on their mapping algorithms: 1) hash table based, seed-and-extend mappers (hash table based mappers) similar to the popular BLAST [21] method, such as mrFAST/mrsFAST [22,23], MAQ [24], SHRiMP [25], Hobbes [26], drFAST [27] and RazerS [28]; and 2) suffix-array and genome compression based mappers that utilize the Burrows-Wheeler Transform and the Ferragina-Manzini index (BWT-FM) such as BWA [29], Bowtie [30], and SOAP2 [31]. Both types of read mapping algorithms have different strengths and weaknesses. To measure the performance of different mappers, three general metrics are introduced: *speed *in performing the mapping, *sensitivity *in mapping reads in the presence of multiple edits (including mismatches, insertions and deletions) and *comprehensiveness *in searching for all mapping locations across the reference genome. The hash table based mappers are much slower, albeit more sensitive, more comprehensive and more robust to sequence errors and genomic diversity than suffix-array based mappers. For these reasons, hash table based mappers are typically more suitable when comparing the genomes of different species, such as mapping reads generated from a gorilla genome to the human reference genome, or when mapping reads to highly repetitive genomic regions where structural variants are more likely to occur [32-34]. On the contrary, suffix-array based mappers (with the BWT-FM optimization) offer very high mapping speed (up to 30-fold faster than hash table based mappers), but their mapping sensitivity and comprehensiveness suffer when the edit distance between the read and the reference fragment is high or when the diversity of the read increases (e.g., when mapping reads from other species). Their fast speed makes the suffix-array based mappers the first choice in single nucleotide polymorphism (SNP) discovery studies where sensitivity is less important. In this work, we focus on increasing the speed of hash table based mappers while preserving their high sensitivity and comprehensiveness.

The relatively slow speed of hash table based mappers is due to their high sensitivity and comprehensiveness. Such mappers first index *fixed-length seeds *(also called *k-mers*), typically 10-13 base-pair-long DNA fragments from the reference genome, into a hash table or a similar data structure. Next, they divide each query read into smaller fixed-length seeds to query the hash table for their associated *seed locations*. Finally, they try to *extend *the read at each of the seed locations by aligning the read to the reference fragment at the seed location via dynamic programming algorithms such as Needleman-Wunsch [18] and Smith-Waterman [17], or simple Hamming distance calculation for greater speed at the cost of missing potential mappings that contain insertions/deletions (indels). For simplicity, the rest of the paper will use the term "k-mer" representing the term "fixed-length seed". We will also use the terms "location" and "seed location" interchangeably.

Using real data generated with the NGS platforms, we observed that most of the *locations *fail to provide correct alignments. This is because the size of the k-mers that form the hash table's indices are typically very short (e.g., 12 bp as default for mrFAST/mrsFAST). These short k-mers appear in the reference genome much more frequently than the undivided, hundreds of base-pair-long query read. As a result, only a few of the locations of a k-mer, if any, provide correct alignments. Naively extending (aligning the read to the reference genome) at *all *of the locations of *all *k-mers only introduces unnecessary computation. In this paper, we define the seed locations that the read cannot align to as "false" locations. Reducing unnecessary computation associated with the large number of false locations is the key to improving hash table based mappers' speed.

In this paper, we propose a new algorithm, FastHASH, that dramatically improves the speed of hash table based algorithms while maintaining their sensitivity and comprehensiveness. We introduce two key ideas for this purpose. First, we drastically reduce the potential locations considered for the extend step while still preserving comprehensiveness. We call this method *Cheap K-mer Selection*. Second, we quickly eliminate most of the false locations without invoking the extend step in the early stages of mapping. This method is called *Adjacency Filtering*. We tested FastHASH by incorporating it into the mrFAST [22] codebase. Our initial CPU implementation of FastHASH provides up to 19-fold speedup over mrFAST, while still preserving comprehensiveness.

In the next section, we describe the basics and the characteristics of Cheap K-mer Selection and Adjacency Filtering. In the Mechanisms section, we present the mechanism of FastHASH in detail. In the Results section, we present the performance of mrFAST with FastHASH compared to the baseline mrFAST and several other read mapping tools. We then present more analysis in the Analysis section and draw conclusions in the Conclusion and Discussion section.

## Observation and insight

### Hash table based mappers

Hash table based mappers map query reads to a known reference genome under a user defined edit distance *e*. With the edit distance *e*, the mappers search for locations where there are fewer than *e *edits (including mismatches, insertions or deletions) between the query read and the reference fragment. Typically, they follow a "seed-and-extend" procedure. These mappers index the reference genome and store the contents in a hash table. The hash table maps all lexicographical permutations of a fixed-length k-mer (typically 10-13 bp) as keys to the corresponding occurrence locations in the reference genome for each k-mer as contents. The indexing procedure is performed only once. During the mapping stage the mapper uses the previously generated hash table to search for seed locations.

**Figure 1:**
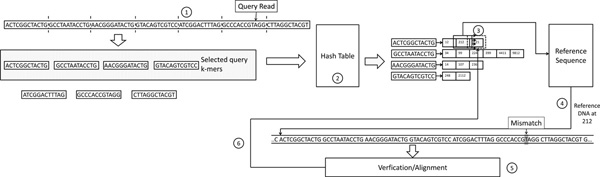
**Hash table based mapping**. The flow chart of hash table based mappers. 1) Divide the query read into smaller k-mers. 2) Search each k-mer in the hash table which is previously generated from the reference genome. 3) Probe location lists. 4) Retrieve the reference sequence starting at the seed location. 5) Align the read against the reference sequence. 6) Move to the next location and redo steps 4 and 5.

Figure 1 shows the flow chart of a typical hash table based mapper during the mapping stage. The mapper follows six steps to map a query read to the reference genome. In step 1, the mapper divides the query read into smaller k-mers, with each k-mer of equal length as the hash table keys. In step 2, several of these k-mers are selected as query k-mers. Query k-mers are then fed to the hash table as inputs. The hash table returns the location lists for each query k-mer. The location list stores all the occurrence locations of the query k-mer in the reference genome. In step 3, the mapper probes the location lists of all k-mers belonging to the query read. For each location, the mapper accesses the reference genome and, in step 4, retrieves the reference fragment from the reference genome at the seed location's neighborhood. In step 5, the mapper aligns the query read against the reference fragment using the Hamming distance or more complicated dynamic programming algorithms such as the edit distance [35], Needleman-Wunsch, or Smith-Waterman, to verify if the number of edits between the query read and the reference fragment exceeds the user-set edit distance *e*. This step is also called the "verification step". One can think of this step as a complicated fuzzy string matching procedure that tries to match the base-pairs between the query read and the reference fragment, with some edits permitted. We will use the term "alignment" or "verification" to refer to this step for the rest of the paper. Finally in step 6, the mapper processes the next location in the location list and repeats step 4 and step 5 until all the locations of the k-mer are processed. This entire process (from step 2 to step 6) is performed for each k-mer in the query read.

### Key observation

Hash table based mappers are computationally more expensive than suffix-array alternatives. Unlike suffix-array based mappers which quickly return the mapping locations at the leaf nodes of the suffix-tree, hash table based mappers try to calculate the optimal alignment for all query k-mers' locations. Mappers that are capable of aligning even in the presence of edits are the most sensitive, yet slowest, since these dynamic programming algorithms typically run in *O*(*l*^2^) time (where *l *is the length of the reads). This can be reduced to *O*(2*el*) if the number of allowed indels are reduced to *e*.

We experimentally tested the behavior of a hash table based mapper mrFAST [22] to identify the performance bottlenecks. We observed that the dynamic programming alignment algorithm (step 5) occupies over 90% of the execution time while most locations fail to pass the alignment verification. Due to the short k-mer size (10-13 bp) and the repetitive nature of most genomes (including human), the location list of a k-mer may contain many locations to which the full query read does not map. Yet the mapper still tries to align the query read to all of the extra locations (step 5) since it has no knowledge of which seed locations provide correct mapping beforehand.

**Figure 2:**
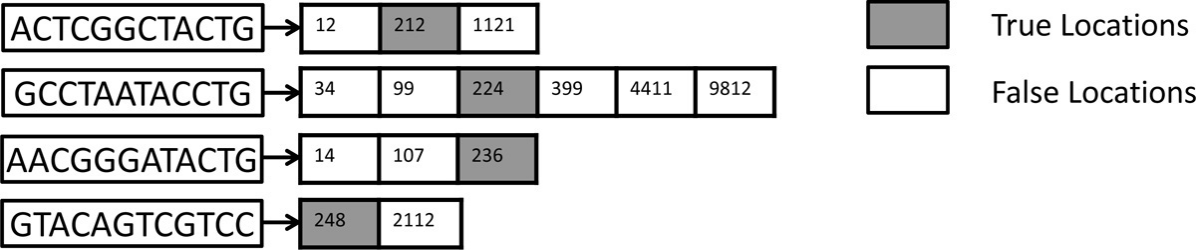
**True vs. false locations**. Example of true locations vs. false locations. Only the true locations provide correct mapping results.

Within a k-mer's location list, we define those locations that pass alignment verification (step 5) as "true locations" and other locations that fail the verification as "false locations". The false locations do not provide mapping results. Figure 2 gives an example of true locations versus false locations. In Figure 2, we have the location lists of the same query read from Figure 1. From the figure, we may conclude that location 212 is more likely to be a "true location", since all k-mers have locations adjacent to location 212 stored in their location lists (e.g., 224, 236 etc.). Other locations are less likely to be a "true location" as they are more isolated (e.g., 1121, 9812, etc.). However, existing mappers do not exploit this observation. As a result, the mapper examines all seed locations while wasting a lot of computation resources on verifying false locations (white blocks in Figure 2).

Verification of the vast number of false locations greatly degrades the performance of the mapper as it consumes a massive amount of unnecessary computation and memory bandwidth. To verify a location, the mapper has to 1) access the reference genome sequence starting at the seed location to get the reference fragment and then 2) invoke a complex programming algorithm to align the query read to the reference fragment. Performing these costly operations for a high number of false locations will only waste computational resources as false locations, by definition, do not provide any valid mappings. Therefore improving the performance of hash table based mappers strongly depends on *efficiently *reducing the number of false locations before the verification step.

### Insight

There are two main directions to ameliorate the computational cost imposed by the false locations. First, one can apply a filter within the seed locations and only extend on "true locations" to reduce unnecessary computation. Second, one can select only the k-mers with low occurrence frequency in the reference as query k-mers to avoid probing long location lists, reducing the number of locations to examine. In this work, we propose two new mechanisms that address both directions.

Our first method aims to filter out the *obviously *false locations. Our observation is that by collecting a common set of adjacent locations from the location lists of all the k-mers, we can quickly distinguish obviously false locations from possibly true locations and skip the unnecessary verification steps for the false locations. The basic idea is as follows: A potential seed location from one k-mer's location list can return a *correct *mapping (under the given edit distance *e*) only if other *adjacent k-mers *of the read are also located at *adjacent locations *in the reference genome (e.g., in Figure 2, location 212 in first k-mer's list, location 224 in the second k-mer's list, location 236 in the third k-mer's location list, and so on). Consequently, by checking if all k-mers have the corresponding adjacent locations stored inside their location lists, we may quickly identify false locations without the alignment step (e.g., in Figure 2, location 1121 from the first k-mer's location list is an easily detectable false location since no other k-mer contains adjacent locations in their location lists). To tolerate edits, at most *e *k-mers are allowed to fail the adjacent location searching step. Otherwise, the number of edits (mismatches and in/dels) between the query read and the reference fragment must be greater than *e*, and thus the location should be rejected before the verification step (step 5). We call this method *Adjacency Filtering (AF)*.

Note that AF does not guarantee correct mappings; instead, it rejects obviously false locations. For computing the actual number, location, and content of edits (including sequence errors) the alignment step (step 5) is still needed. Nevertheless, AF detects a large fraction of the false locations (more than 99% on average based on our empirical evaluations) and removes them from consideration for verification.

**Figure 3:**
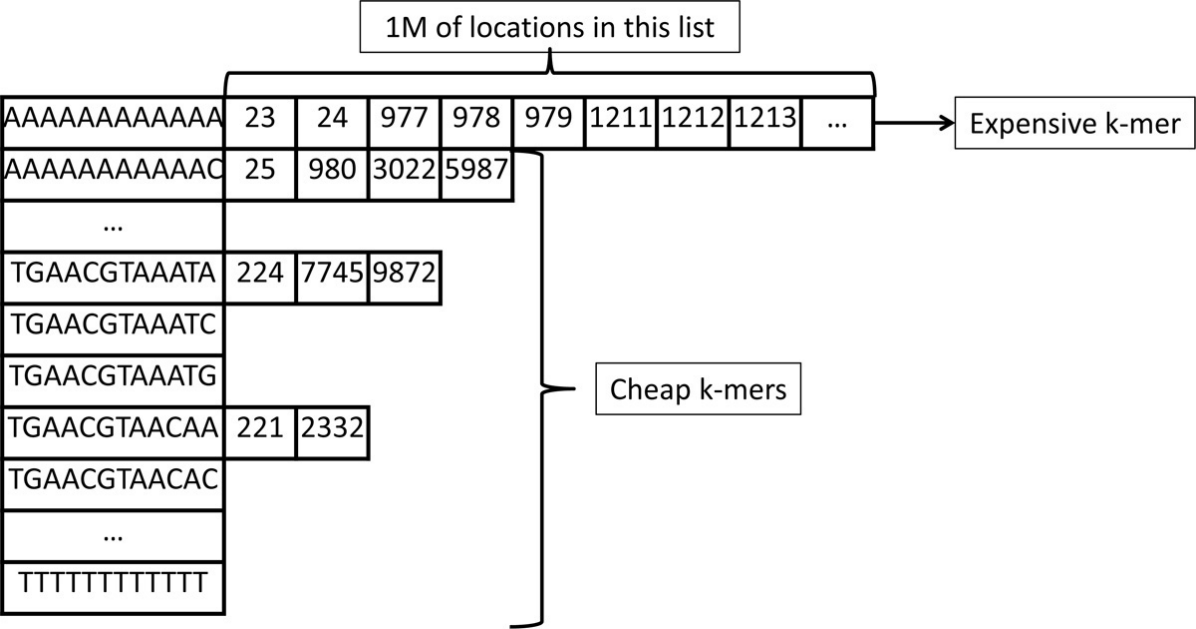
**A example of imbalanced hash table entires**. A snapshot of the hash table. Some k-mers have very large location lists, while others have much shorter lists. For example, AAAAAAAAAAAA has over one million entries into reference genome whereas TGAACG-TAACAA has only 2.

Our second method, *Cheap K-mer Selection (CKS) *tries to minimize verification operations by preferentially selecting and using as seeds those k-mers from the query reads that occur infrequently in the reference genome. For a query read, the amount of alignment computation (step 5) is proportional to the number of locations stored in the location lists of the query k-mers. We observed that selecting different k-mers to query the hash table may heavily affect the mapper's performance, since the reference hash table is heavily unbalanced. Due to the repetitive nature of most genomes and the very short k-mer length, some k-mers have very large location lists (called high frequency k-mers) and others have much smaller location lists (called low frequency k-mers), as Figure 3 shows. Probing large location lists burdens the mapper since it has to verify a large number of locations; thus, we call these high frequency k-mers as *expensive k-mers*. On the other hand, k-mers with smaller location lists are denoted as *cheap k-mers*. Our insight is that, for a correct mapping, both cheap and expensive k-mers have the true locations stored in their location lists. However, expensive k-mers have several orders of magnitude more false locations than cheap k-mers, due to their repeating nature in the reference genome. As a result, selecting cheap k-mers instead of expensive ones as query k-mers reduces the number of locations to be verified (steps 3 to 6) without affecting the mapper's sensitivity. Sensitivity is guaranteed by selecting multiple cheap locations to ensure that their combined coverage includes all possible editing scenarios (having no more than *e *mismatches, insertions or deletions. e.g., in Figure 2, when *e *= 3, by selecting four non-overlapping cheap k-mers, we ensure finding all mappings with at most three edits since three edits can alter at most three k-mers).

With AF eliminating unnecessary computation to detect false locations and CKS reducing the number of false locations, our new algorithm, FastHASH, is able to minimize unnecessary computation and focus on mapping only at possible true locations, which provides drastic speedup over previous hash table based mappers, as our experimental results show.

## Mechanisms

### Adjacency filtering (AF)

Adjacency Filtering (AF) uses the location lists retrieved from the hash table to detect false locations. Since the location lists are stored contiguously as sorted arrays in the hash table, it is easy to prefetch these lists into the CPU cache. Moreover, the location lists exhibit high temporal locality. Once fetched into the cache, the location lists of the k-mers can be used to verify all seed locations, and thus will be reused many times. Unlike traditional hash table based mappers which try to extend at all potential seed locations and perform many unpredictable reference genome lookups, FastHASH with AF only accesses the reference genome when it is confident that the seed location is very likely to be a true location.

**Figure 4:**
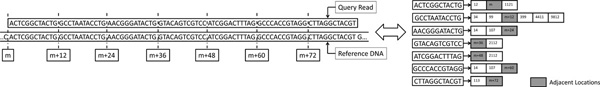
**An example of Adjacency Filtering**. The insight behind adjacency filtering: For a perfect mapping, all adjacent k-mers within a read should also be at adjacent locations within the reference. This is equivalent to searching for adjacent locations in adjacent k-mers' location lists.

Briefly, FastHASH divides a read into consecutive k-mers and tests whether k-mers that are adjacent to each other within the read are also found at adjacent positions within the reference. For example, let us assume that the size of the k-mers in the hash table is *k *= 12, the length of the query read is 84 base-pairs (bp), and the mapper's edit distance is set to *e *= 0, which allows no edits. The mapper first divides the read into 7 *consecutive *k-mers of length 12 bp each, and then uses the locations of these k-mers in the hash table as seeds. As the left half of Figure 4 shows, for a true location *m *(where the query read perfectly maps to the reference), the first k-mer of the read is at location *m*, the second k-mer is at *m *+ 12, third k-mer is at *m *+ 24; and this pattern continues up to the 7*^th ^*k-mer, which is located at *m *+ 72. Similarly, if *m *is an unknown seed location and we want to know whether the location is a true location or false location, we can simply verify whether *m *is stored in the first k-mer's location list, *m *+ 12 in the second k-mer's location list, and so on, as shown in the right half of Figure 4. Here, we define k-mers for which we can and adjacent k-mers at adjacent locations as *correct k-mers*, and others as *edited k-mers*. Now suppose that the read contains some edits from the reference fragment; then such edits must affect at least one k-mer, which in turn alters the k-mer to be different from the reference fragment, becoming an edited k-mer. As a result, the corresponding adjacent location will not show up in the location list of the edited k-mer. By simply testing if all the corresponding adjacent locations are present in all of the adjacent k-mers' location lists, we can detect edits without actually aligning the read to the reference fragment.

**Figure 5:**
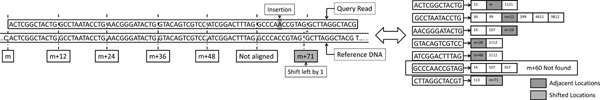
**An example of Adjacency Filtering with errors**. An example of insertion tolerance. Because of an insertion on the 6*^th ^*k-mer, the 6*^th ^*k-mer becomes an edited k-mer and the mapper cannot find this k-mer's adjacent location. Even worse, the insertion also shifts all down stream k-mers to the left by 1-bp. However the 7*^th ^*k-mer is still considered as a "correct k-mer" since it has location *m+71*, which is in the adjacent range [m+71, m+73], in its location list.

If some finite number of edits are allowed, for example up to a total of *e *mismatches, then it is possible that a location still provides a correct mapping with at most *e *edited k-mers (as in the worst case, each edited k-mer contains only one edit). Here we explain how AF provides edit tolerance in two steps: We first explain how AF handles mismatches and then describe how insertions and deletions are handled. With at most *e *mismatches, in the worst case (i.e., when the mismatches are spread across *e *k-mers), a mapping location can still lead to a valid mapping with at most *e *k-mers failing the adjacent location test. In essence, to incorporate *e *mismatches into AF with a read divided into *N *k-mers, we require at least *N *- *e *k-mers with corresponding adjacent locations in their location lists. Otherwise, the location is marked as a false location and rejected before further operations. Allowing insertions and deletions is similar but requires a little bit more analysis. Additionally to the above observation, an insertion or deletion not only fails the searching of the adjacent location for the edited k-mer, but also shifts all the downstream k-mers as well, as shown in Figure 5. In the presence of insertions or deletions, the AF requirement is further relaxed from requiring searching for a single adjacent location to searching for an adjacent range. For example, if the user allows one insertion/deletion, then instead of searching location *m *in the first k-mer's location list, we now search for locations [*m *- 1, *m *+ 1] in the first k-mer's location list, [*m *+ 11, *m *+ 13] in the second k-mer's location list and so on. To sum up, with at most *e *edits (mismatches, insertions or deletions), a potential location passes AF only if *N *- *e *k-mers find corresponding adjacent location ranges within their location lists, with adjacent range defined as [-*e*, +*e*] deviation range from the adjacent location. Otherwise, the location is marked as a false location and the mapper moves to the next location (step 3).

The power of AF comes from detecting and rejecting most of the false locations before verification. Not only does AF prevent unnecessary computation (step 5), but it also prevents expensive unnecessary memory accesses to the reference genome to retrieve the reference fragment (step 4). For a false location, other than the query k-mer itself, usually the rest of the read is completely different from the reference genome. We observed in real data sets that even with high edit distance *e *= *5*, only a small fraction (usually less than 1%) of the locations pass AF and are marked as potential true locations for further verification. As a result, AF is effective at detecting and rejecting false locations.

However, AF also comes with its own computational cost. To test a potential location, AF conducts *N *searches for adjacent locations, one for each k-mer's location list. Additionally, AF does not guarantee that the remaining seed locations will have fewer than *e *edits after alignment, since multiple edits might reside in a single edited k-mer. In such cases, AF will not be able to tell exactly how many edits there are, so it conservatively assumes there is only one edit per edited k-mer and passes the location to the verification step (step 5). During the alignment step, a dynamic programming algorithm extracts detailed editing information and verify if the mapping is indeed correct. To summarize, for true mapping locations (with fewer than *e *edits), AF introduces extra computation. Nevertheless, AF is cost-efficient because the number of the locations that pass AF is marginal compared to the number of locations that are correctly filtered out.

### Cheap K-mer selection (CKS)

Although AF reduces memory lookups, it also incurs a penalty in detecting false locations: AF searches the corresponding adjacent locations for every k-mer. This is in fact a quick lookup in the location lists: as the location lists are sorted, we can use binary search. Nevertheless, for longer reads with many k-mers, AF can be a costly process. From our experiments, we see that AF reduces the alignment calculation (step 4 and 5) by over 90% but provides only 2x speedup. After profiling the execution, we observe that AF has become the new bottleneck by occupying over 90% of the execution time.

The core problem stems from the imbalance of the hash table. Most location lists in the hash table for the human genome have very few locations. However, there are also location lists with cardinality greater than 1 million. Even though such k-mers are only a small portion of the hash table, we encounter them frequently with real data. These high frequency (or expensive) k-mers mostly correspond to poly-N tracks and microsatellites [36], and such sequences have many copies in the human reference genome. These expensive k-mers also introduce many false locations. When we use such expensive k-mers to query the hash table, all of the locations in their entries will go through the AF test, which is a search-heavy (i.e., computationally expensive) process.

FastHASH actively selects *cheaper *k-mers over more expensive k-mers as query k-mers. There will be fewer false locations and fewer invocations to AF with cheaper query k-mers. Note that, for any read, both cheap and expensive k-mers will have the same true locations in their location lists. However, since by definition expensive k-mers are more frequent in most genomes, including the human genome, they will contain substantially more seed locations than cheaper k-mers, thus imposing more computational cost to both AF and the subsequent verification step. Instead, starting the AF and then the alignment with the cheap query k-mers relieves the mapper of this cost while preserving all the true locations.

We implemented the selection of cheap k-mers as a simple quicksort operation before querying the k-mers in the hash table. For each query read, instead of simply selecting the first *e+1 *k-mers to search in the hash table, FastHASH first sorts all k-mers by the cardinalities of their location lists, and then selects the cheapest *e+1 *k-mers (i.e., those k-mers that have the smallest cardinality of their location lists). Note that selecting *e+1 *k-mers as query k-mers guarantees full sensitivity under edit distance *e*.

In summary, Cheap K-mer Selection (CKS) reduces the number of AF and verification operations by using a computationally cheap operation: quicksort. There are other mechanisms to further reduce the number of the false locations with more complex computation and more memory accesses, as done by Ahmadi et al. [26]. In their work "Hobbes", instead of simply dividing the read contiguously, Ahmadi et al. test all possible ways of dividing a query read into multiple k-mers and select only the cheapest division (the way of dividing the query read that returns the cheapest set of k-mers). However, to assess the cost of one possible division of the query read into k-mers, Hobbes has to access the hash table multiple times to get the location list length for each k-mer belonging to the division. We believe Hobbes may not be cost-efficient compared to CKS for two reasons: 1) With CKS, the query k-mers are already very cheap. From our observation, most of the query k-mers have fewer than ten locations after CKS. 2) Hobbes incurs tens to hundreds of accesses to the hash table with only slightly cheaper query k-mers than CKS. The benefit of having a slightly cheaper query k-mer set is very likely offset by the cost of having many long latency memory accesses to the hash table. In fact, CKS avoids examining a lot of the false locations with very few memory accesses (O(log *N*) accesses, where *N *is the number of k-mers to which a query read can divide).

## Methodology

We implemented FastHASH on top of mrFAST version 2.1.0.6, creating a new version, mrFAST-2.5.0.0. To assess the performance of FastHASH, we compared the performance of the new mrFAST-2.5.0.0 against several popular read mappers currently available including Bowtie, BWA, RazorS and mrFAST-2.1.0.6, both on simulated and real data sets. We evaluated the mappers with respect to three metrics: *speed, sensitivity *and *comprehensiveness*. We also tried to benchmark Hobbes but its very large memory footprint resulted in page thrashing, greatly degrading performance. As a result, we do not report the performance results of Hobbes.

*Speed *is how fast a mapper maps reads to the reference genome, and is defined as the execution time of the binary measured by the Linux utility "time". *Sensitivity *is defined as the fraction of reads where the mapper finds at least one mapping. A higher mapper sensitivity correlates to an improved ability to tolerate edits. *Comprehensiveness *is how many true locations the mapper finds for a given read. A higher mapper comprehensiveness correlates to a more thorough ability to search the reference genome.

We tested speed, sensitivity and comprehensiveness with different edit distances *e *from 1 to 5 for all mappers with three real data sets. Then, we mapped three simulated data sets with a fixed edit distance 3. Since Bowtie does not support any edit distance greater than 3, we only have results for Bowtie with edit distances 1, 2 and 3. RazerS supports edit distance via a percent identity setting. In order to provide fair comparison, we chose the edit percentage as close to the edit distance as possible. For simulated reads, we guarantee each read contains at most 3 edits from the reference genome. As a result, a fully sensitive mapper should be able to map all simulated reads with edit threshold greater than or equal to 3.

*Real Data: *We used three different real data sets to evaluate the performance of different mappers. All sequence data sets were generated using the Illumina platform. The first set (set 1; 160 bp per read, 1 million reads) consists of reads from an individual obtained from the 1000 Genomes project [2] sequenced with the Illumina platform. The second set (set 2; 101 bp per read, 500,000 reads) is generated from a chimpanzee genome [37], and the third set (set 3; 70 bp per read, 500,000 reads) is generated from an orangutan genome [38]. In our benchmarks, we mapped all reads to the current human reference genome assembly (GRCh37, hg19).

*Simulated Data: *We generated three simulated data sets from the current human reference genome assembly (hg19). For each set, we generated 50,000 random reads from the first 20 chromosomes summing up to 1 million reads for each set. The sets differ in their read lengths: 72 bp, 108 bp, and 180 bp. For each read, we simulated the read errors and edits by randomly altering or inserting/deleting 0 to 3 base-pairs. Each set is mapped to the human reference genome (hg19).

We ran all mappers in single user mode on a Linux machine with a 3.2 GHz Intel i7 Sandy Bridge CPU and 16 GB DDR3-1333 main memory.

## Results

As Additional file 1 shows, hash table based mappers such as mrFAST-2.1.0.6 and RazerS suffer from low performance (slow speed) compared to BWA and Bowtie. As we will show in the "Analysis" section, this is mainly due to the massive amount of false locations. FastHASH (mrFAST-2.5.0.0) greatly improves mapping speed over mrFAST-2.1.0.6 (e.g., up to 19 times for *e *= 3, depending on the data set), and is even faster than BWA under certain circumstances (e.g., for set 1, when edit distance is greater than 3). Meanwhile, FastHASH preserves the important sensitivity and comprehensiveness properties of the previous version of mrFAST, mrFAST-2.1.0.6.

**Figure 6:**
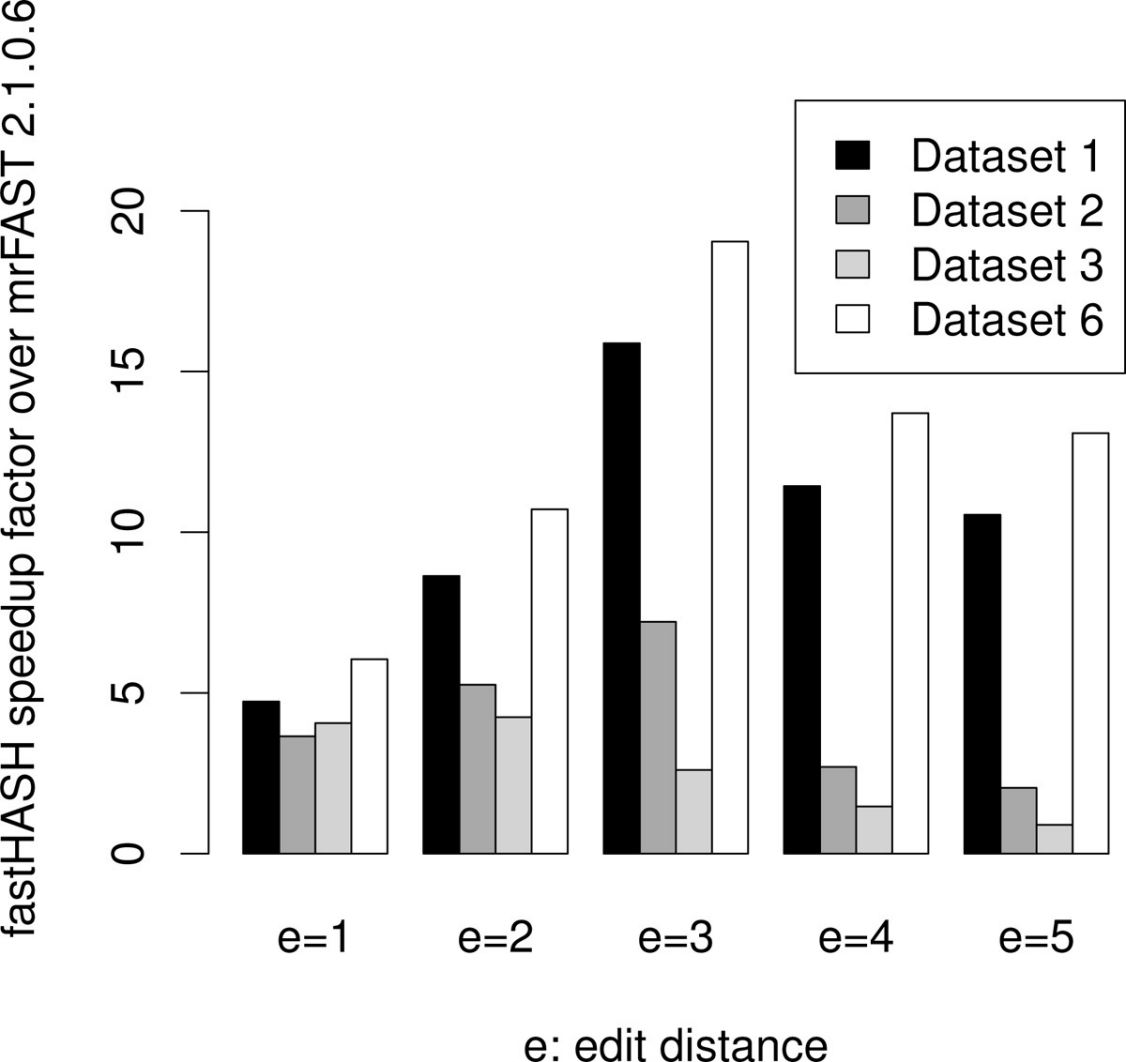
**Speedup of FastHASH**. Speedup factor of FastHASH (mrFAST-2.5.0.0) over mrFAST-2.1.0.6, with different read sets.

Figure 6 presents the speedup of FastHASH (mrFAST-2.5.0.0) over mrFAST (mrFAST-2.1.0.6) across different edit distance values on different data sets. Notice that as edit distance *e *increases, the speedup decreases. This is expected since a higher *e *results in diminished CKS benefits. We provide further details in the "Analysis" section.

Table 1 shows the sensitivity of different mappers on simulated data sets. For a simulated data set, since all the reads are generated from the reference human genome and are guaranteed to have fewer than 3 edits (mismatch, insertion or deletion), an ideal mapper should be able to map all 1 million reads. In reality, due to performance constraints or simply mapping limitations, most mappers do not guarantee full sensitivity. mrFAST on the other hand, achieves 100% sensitivity. We clearly see that mrFAST with FastHASH retains 100% sensitivity of mrFAST-2.1.0.6 on simulated data sets. In fact, since mrFAST-2.5.0.0 includes several minor bug fixes, the sensitivity is slightly higher than the earlier mrFAST, mrFAST-2.1.0.6. Note that a higher *e *always leads to more mappings and should intuitively be slower. However, for some input sets, FastHASH counter-intuitively runs faster for higher edit distances than lower edit distances. This is because mrFAST uses Intel SSE SIMD code extensions [39] which marginally alters the mapper algorithm used based on the edit distance. In particular, the algorithm for *e *= 4 is slightly faster than that for *e *= 3. Generally, however, all mappers are slower with higher edit distances as expected. We also show further analysis in the "Analysis" section.

**Table 1:** Simulated Set

Data Set	Mapper	Time (min.:sec.)	Reads Mapped	Map Locations
Set 4	*mrFAST-2.5.0.0*	158:13	1000000	112638835
	mrFAST-2.1.0.6	531:48	1000000	112638623
	Bowtie-0.12.8	27:12	831211	95923952
	BWA-0.6.1	35:55	978102	65489552

Set 5	*mrFAST-2.5.0.0*	30:38	1000000	26957339
	mrFAST-2.1.0.6	455:40	1000000	26957196
	Bowtie-0.12.8	14:47	747457	22039633
	BWA-0.6.1	30:35	952953	23468560

Set 6	*mrFAST-2.5.0.0*	19:34	1000000	4484323
	mrFAST-2.1.0.6	380:28	1000000	4484055
	Bowtie-0.12.8	12:07	614827	3303329
	BWA-0.6.1	24:34	883520	4427109

Comparison of mapping sensitivity across the selected mappers using three simulated reads (Sets 4, 5 and 6) with read lengths of 72, 108 and 180 basepairs respectively. Each benchmark is referenced against the human reference genome using an edit distance of 3.

**Figure 7:**
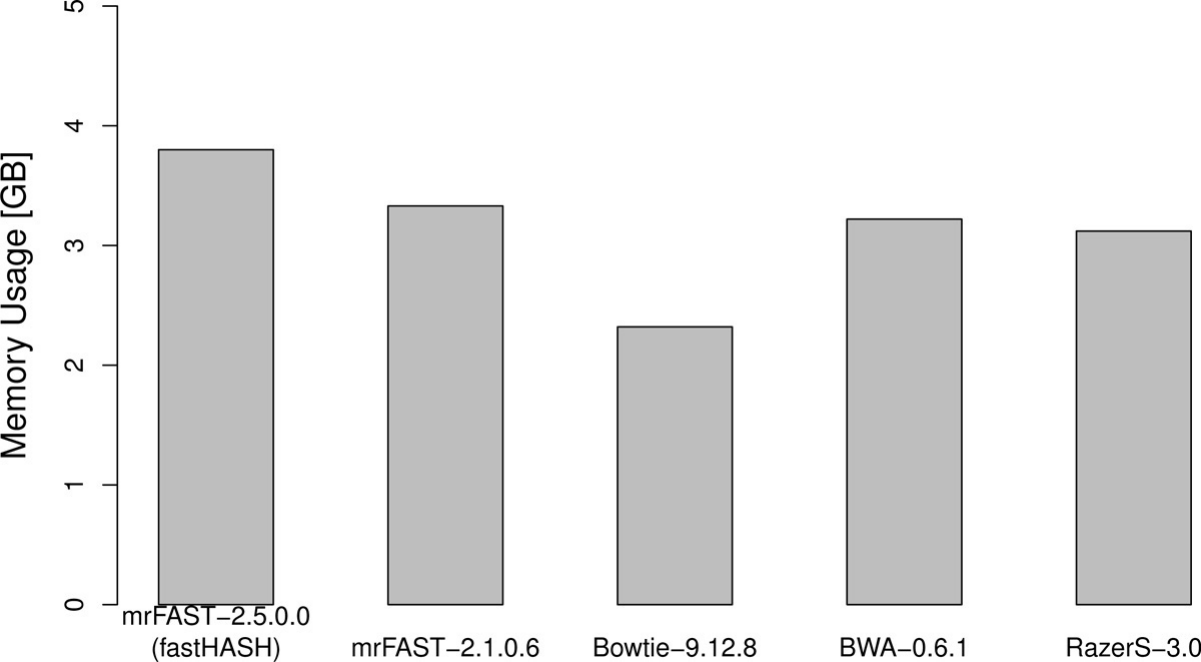
**Memory usage of different mappers**. Memory usage comparison across different popular mappers.

Figure 7 shows that the memory usage of mrFAST-2.5.0.0 does not change significantly compared to mrFAST-2.1.0.6.

## Analysis

**Figure 8:**
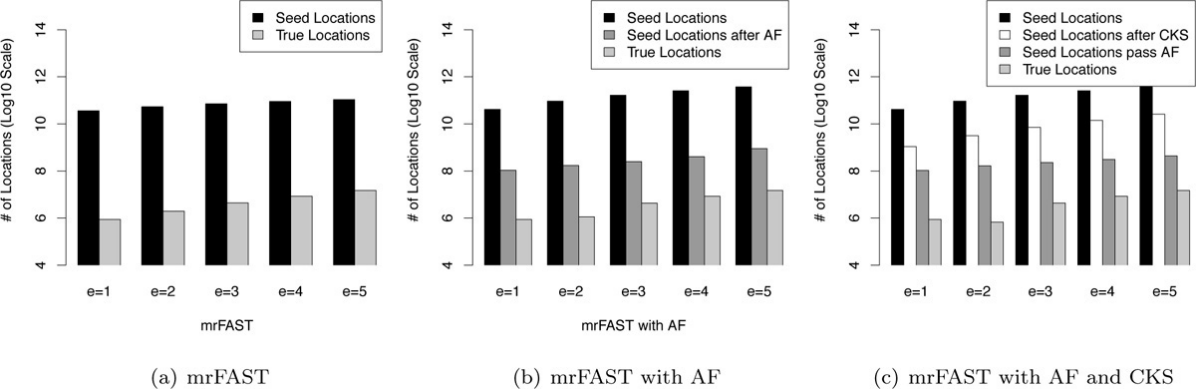
**Improvement breakdown of AF and CKS**. Breakdown of incremental improvement by AF and CKS. a) mrFAST, b) mrFAST with AF, c) mrFAST with AF and CKS.

In this section, we analyze the benefits of Adjacency Filtering and Cheap K-mer Selection. The benefits are shown in Figure 8 (note that the y-axis is logarithmically scaled).

As discussed in the previous section, mrFAST-2.1.0.6, like other hash based mappers, suffers greatly from extending on a large number of false locations. Figure 8(a) presents the number of true locations out of all potential locations: only 0.007% of the potential locations (seeds) provide correct alignment on average.

We can clearly see the incremental benefits of AF and CKS when mapping 1 million simulated reads of 180 bp in length (Figure 8(b) and Figure 8(c)). As discussed above, a very small fraction of the seed locations pass the verification step of mrFAST (Figure 8(a)). Adjacency filtering substantially decreases the number of seed locations by eliminating false locations as seen in Figure 8(b). This way, AF saves many unnecessary memory accesses since now only the locations that pass AF will proceed to further verification. On average, AF filters out approximately 99.8% of all false locations. Figure 8(c) shows the benefit of having both AF and CKS. From Figure 8(c), we see that CKS reduces the number of overall potential locations, which reduces the amount of AF computation. On average, CKS eliminates 95.4% of all seed locations without degrading the sensitivity of the mapper (as seen in Table 1).

**Figure 9:**
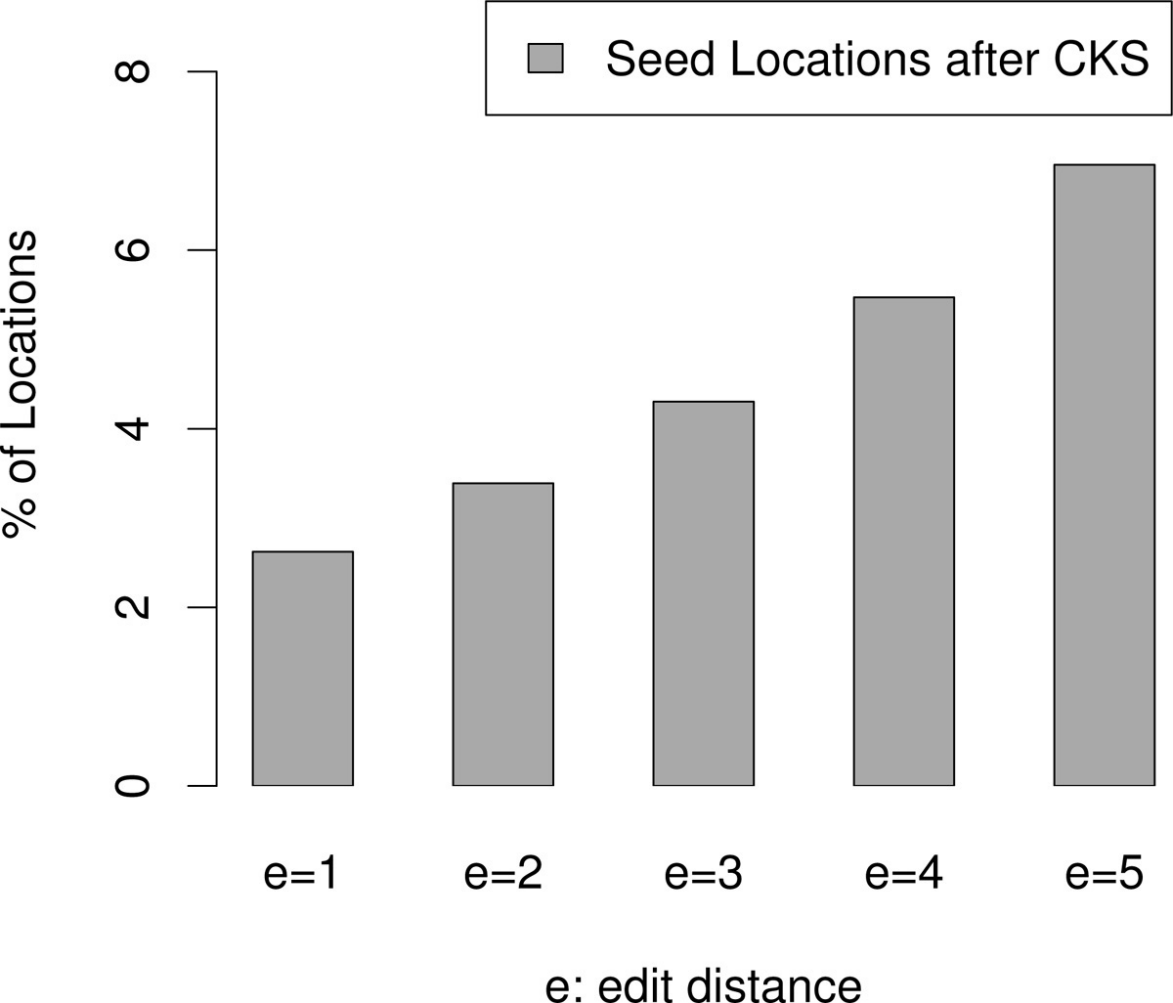
**Effectiveness of CKS across different edit distances**. The figure shows the fraction of the seed locations that pass CKS. Lower values are better. As the edit distance increases, CKS becomes less effective since a larger fraction of locations pass CKS.

In the Result section, we showed that the speedup gained by using FastHASH reduces as edit distance increases. This is because as *e *increases, CKS starts to select more expensive k-mers, as Figure 9 shows, providing less reduction of false locations.

## Conclusions and discussion

Next generation sequencing platforms continue to evolve at a fast rate. New technologies are frequently introduced that offer different strengths; each, however, has unique biases. The current trend is to generate longer reads, with newer technologies such as the nanopore sequencing, at the cost of increased error rates. While the sux-array based mappers offer tremendous read mapping speed, they also suffer greatly with higher error rates and longer read lengths. Seed-and-extend hash table based mappers are more robust to these changes, but they are also very slow for mapping short reads.

In this paper, we analyzed seed-and-extend, hash table based read mapping algorithms and proposed a new optimization algorithm called FastHAST that provides up to 19-fold speedup over the best previous seed-and-extend mapper. FastHASH provides a potential solution to the speed inefficiency problem of hash table based mappers as a generic algorithm that can be implemented in with any such read mapper.

Although our current implementation of FastHASH is on a CPU based system, we also have a preliminary implementation on a GPU based system, which we aim to develop further. Another future direction to improve FastHASH is to develop a hybrid indexing strategy that efficiently merges Burrows-Wheeler Transform and the Ferragina-Manzini indexing with FastHASH to increase seed size for longer (*>*1 kbp) reads while keeping the memory footprint low.

Together with additional GPU-based improvements for the alignment step of read mapping, FastHASH promises to accelerate read mapping further while maintaining the sensitivity of hash table based mappers to help cope with the overwhelming data deluge created by next generation sequencing.

## Competing interests

The authors declare that there are no competing interests.

## Authors' contributions

HX and DL designed the FastHASH algorithm. HX, DL, and FH implemented the described methods. SY performed comparisons and helped HX and DL perform the analyses. OM and CA conceived and planned the experiments, and supervised HX and DL for the algorithm development. All authors contributed to the writing of the manuscript.

## Declarations

The publication costs for this article were funded by the NIH grant HG006004 to O.M. and C.A.

This article has been published as part of *BMC Genomics *Volume 14 Supplement 1, 2013: Selected articles from the Eleventh Asia Pacific Bioinformatics Conference (APBC 2013): Genomics. The full contents of the supplement are available online at http://www.biomedcentral.com/bmcgenomics/supplements/14/S1.

## Supplementary Material

Additional file 1 **Performance on Real Data Sets**. Performance comparison between different methods, while using three different data sets. Set 1 is a set of 1 million reads of length 160 bp obtained from a human genome sequenced within the 1000 Genomes Project. Set 2 is composed of 500,000 reads of length 101 bp generated from a chimpanzee genome, and Set 3 is from an orangutan genome with reads of length 75 bp (500,000 reads). We select edit distance values from 1 to 5 in order to compare the speed (time), sensitivity (Reads Mapped) and comprehensiveness (Map Locations) of each mapper. Click here for file
